# The association between violent video game exposure and sub-types of school bullying in Chinese adolescents

**DOI:** 10.3389/fpsyt.2022.1026625

**Published:** 2022-11-16

**Authors:** Yuhang She, Zidan Yang, Lingyu Xu, Liping Li

**Affiliations:** ^1^School of Public Health, Shantou University, Shantou, China; ^2^Injury Prevention Research Center, Shantou University Medical College, Shantou, China

**Keywords:** school bullying, violent video game, association, adolescents, China

## Abstract

**Background:**

School bullying among adolescents has been a worldwide public health issue. It has been observed that adolescents who are exposed to violent video games (VVGs) are often more aggressive. However, research on the association between violent video game exposure (VVGE) and different types of school bullying is limited in the Chinese context.

**Objective:**

The purpose of this study was to explore whether VVGE is linked to school bullying behaviors among Chinese adolescents and to examine the relationship between different levels of violent game exposure and four sub-types (physical, verbal, relational, and cyber) of school bullying involvement.

**Methods:**

This was a cross-sectional study of 1,992 Chinese students (55.02% boys and 44.98% girls) with the average age of 15.84 ± 1.62 years. Sub-types of school bullying victimization and perpetration, Internet addiction, and VVGE were measured by using a self-administrated questionnaire. The association was examined by multiple logistic regression analysis, adjusting for covariates.

**Results:**

Physical, verbal, relational, and cyber school bullying victimization were reported by 18.12, 60.34, 11.75, and 12.05% of the adolescents, and physical, verbal, relational, and cyber school bullying perpetration were reported by 16.62, 54.62, 21.49, and 8.23% of them. Of the students, 1,398 (70.18%) were normal Internet users, 514 (25.80%) showed moderate Internet addictive behaviors, and 31 (1.56%) of the students showed severe Internet addictive behaviors. The prevalence of no VVGE, low-level VVGE, medium-level VVGE, and high-level of VVGE were 27.70, 24.10, 24.20, and 24.00%, respectively. The risk of physical victimization and physical perpetration significantly increased with the increasing degree of violent video game exposure (*P* for trend < 0.001), with the highest adjusted odds ratios (ORs) of 2.251 (95% CI 1.501–3.375) and 2.554 (95% CI 1.685–3.870), when comparing high-level VVGE with no VVGE.

**Conclusion:**

These findings highlight the specific association between different sub-types of school bullying involvement and violent video game exposure. Physical school bullying prevention and intervention programs should be conducted after adolescents are exposed to violent video games.

## Introduction

School bullying has been a worldwide public health issue. The prevalence of school bullying among adolescents has been reported to vary from 5.1 to 45.1% worldwide (1–3). School bullying is defined as a form of school violence that is performed repeatedly and intentionally against other students through the imbalance of power between the perpetrators and the victims occurring in the context of school settings (4). Traditionally, direct school bullying includes physical (e.g., hitting, kicking, pushing, and shoving around) and verbal (e.g., teasing and name-calling) behaviors, as well as indirect forms of intentional social isolation such as relational (e.g., spreading rumors and manipulating friends' relationships) behaviors (4, 5). In addition, the undeniable degree of youth's access to digital communications leads to the emergence of cyberbullying, which is described as willful and repeated harmful actions to the victims through the use of electronic devices such as computers and cell phones (6).

Likewise, previous studies reported that the prevalence of traditional bullying was 35.3%, and over 31.0% of Chinese adolescents have experienced cyberbullying, suggesting that school bullying is also a significant health problem in China (1). Studies also found that adolescents who experienced cyberbullying were more likely to report experiencing traditional bullying (7). Sometimes, the occurrence of cyberbullying overlapped with traditional bullying, and adolescents who faced both traditional and cyberbullying suffered a higher level of adverse health-related symptoms than those who were involved with a single form of bullying (8).

With the rapid development of the Internet and mobile electronic devices (e.g., smart phones and tablets), video games, as one of the important ways of leisure and social connection, have become more and more popular among teenagers in China. According to the reports of the China Internet Network Information Center (CNNIC) in 2021, the number of underage (younger than 18 years) Internet users in China has continued to grow, and the prevalence rate of Internet usage among underage people was as high as 94.9%. Moreover, 82.9% of the underage people had their own Internet access devices, and 62.5% of them were video game players, of which middle school and high school students accounted for more than 70% (9). However, to keep attracting players into games, game merchants have been adding violence and other elements into games, leading the raised potential concern of problematic engagement in youth game playing (10).

Violent video games (VVGs) are those that depict intentional attempts by individuals to inflict harm on others in video games (11). Having exposure to VVGs has been shown to increase aggression in players, which could also be transmitted through players' social networks, leading to the social networks of players exposed to VVGs becoming more aggressive as well (12). A correlation analysis also showed that VVGs were significantly and positively associated with online aggressive behaviors (13). A cross-sectional study among Iranian adolescents showed a relationship between exposure to VVGs and adverse mental health effects, with symptoms such as increased problematic behavior and deterioration of mental health among excessive game players, such as anxiety, social dysfunction, and depression (14).

Studies on school bullying or adolescents who had been exposed to violent video games were separated, and little is known about the association between violent video game exposure (VVGE) and different types of school bullying among adolescents. Researchers found that adolescents who were exposed to a greater amount of violent video games were more hostile and more involved in physical fighting (15). Previous cross-sectional studies, longitudinal studies, and meta-analyses also showed that violent video game exposure could predict adolescents' aggressive behavior (11, 16, 17). However, most of the studies on violent video game exposure were conducted in Western countries, and there is limited research examining the relationship between VVGE and different types of school bullying involvement among Chinese adolescents.

Thus, our study aimed to explore whether VVGE was linked to school bullying behaviors among Chinese adolescents, and we also evaluated the relationship between different levels of violent game exposure and four sub-types (physical, verbal, relational, and cyber) of school bullying involvement (victimization and perpetration) to provide a specific view of the school bullying prevention program for school policymakers from VVGE perspectives. We hypothesized that (1) the incidence of school bullying among Chinese adolescents is quite common, (2) violent video games are popular among Chinese adolescents, and (3) all four sub-types of school bullying behaviors were associated with VVGE, and adolescents who did not play VVGs were less likely to be involved in school bullying behaviors.

## Methods

### Participants and procedures

This was a cross-sectional study conducted in June 2021 in Shantou, Guangdong Province, which is located in southeastern China. By using the stratified cluster sampling method, Shantou was divided into two areas: downtown and suburban, from which two middle schools and two high schools were selected separately.

The inclusion criteria were as follows: (a) students from the selected schools and (b) students who signed the consent forms. The exclusion criteria were as follows: (a) students who were absent from school during the survey procedure, (b) students older than 18 years, (c) students who did not complete the survey, and (d) students in the 9th grade and 12th grade due to the preparation of entrance examinations.

Before the study, every student was informed about the purpose and assured the anonymity and confidentiality of the study before they filled questionnaires by our trained researchers, and the students were also allowed to discontinue at any time if felt discomfort in the study. After signing the consent forms, questionnaires were distributed to each student. The students were asked to complete the questionnaires during a 20- to 30-min-long class time. This study was approved by the Ethics Committee of Shantou University Medical College.

We distributed the questionnaires to 2020 students. After excluding 28 invalid questionnaires that were unfinished or finished but with too much missing data, a total of 1,992 participants were finally eligible for enrollment in the study. The responding rate of this study was 98.61%.

### Measurements

#### Demographic variables

Demographic variables were gender (boys or girls), grade (7^*th*^, 8^*th*^, 10^*th*^, and 11^*th*^), smoking status (yes or no), drinking status (yes or no), number of friends (1 or less, 2–3, or more than 3), family income (poor, average, or rich), and only child (yes or no). Family structure was divided into three categories: two-parent family, single-parent family, and reconstituted family. Family members' relationship was classified into three categories: harmony, general, and always conflict. Relationship with parents was divided into three categories: never fight, occasional fight, and always fight. The frequency of communication with parents was classified into three categories: seldom, occasional, and regular.

#### Internet addiction

We used the Internet Addiction Test (IAT) scale to assess Internet addiction of the participants. The IAT scale, developed by Kimberely S. Young in 1988, comprised 20 items scored on a five-point Likert scale ranging from 1 to 5 (with 1 denoting rarely and 5 denoting almost). The higher the total score, the greater the degree of Internet addiction of the participants. According to the different total scores, the degree of Internet addiction was divided into the following three categories: normal Internet users (20–49 points), moderate addictive behaviors (50–79 points), and severe addictive behaviors (80–100 points). A total score ≥50 indicates Internet addiction (18). The main contents of the scale included problems related to the excessive use of the Internet, such as ignoring their work and surfing the Internet, spending more time on the Internet because of low self-control, and lying to family members to conceal the extent of usage of the Internet (19). Studies showed that the IAT scale has good reliability and validity in different populations across different countries (20, 21). In this study, Cronbach's alpha was 0.993.

#### School bullying victimization and perpetration

We used the Juvenile Campus Violence Questionnaire (JCVQ) to access all the sub-groups of school bullying victimization and perpetration, which had high reliability and validity in the use of school violence behaviors in Chinese adolescents (Cronbach's alpha was 0.905) (22). The JCVQ included domains of physical and psychological school violence, consisting of 36 items on victimization and perpetration covering all the sub-groups of school bullying. Each student was asked about how often these items occurred over the past year. The frequency of each item was scored on a four-point Likert scale ranging from 1 to 4 (with 1 denoting never, 2 denoting sometimes, 3 denoting often, and 4 denoting almost). The participants were categorized as involved or not involved in each form of school bullying victimization or perpetration if the frequency code was > 2. Cronbach's alpha in this study was 0.954.

In this study, we measured four sub-types of school bullying behaviors *via* four dimensions of the JCVQ. Physical bullying victimization was measured by the following items of physical aggression dimension: (1) having been hit, kicked, pushed, or shoved and (2) having belongings been taken or damaged. Verbal bullying victimization was measured by the following items of verbal aggression dimension: (1) having been called nasty names and (2) having been made fun of. Relational bullying victimization was measured by the following items of the relational aggression dimension: (1) having been excluded from the group or completely ignored and (2) having been spread rumors or having been disliked by others. Cyberbullying victimization was measured by the following items of cyber violence dimension: (1) having been called nasty names or made fun of online and (2) having been spread rumors online. The JCVQ was also used to assess the sub-types of school bullying perpetration by using the same patterns mentioned before.

#### Violent video game exposure

The violent video game exposure questionnaire developed by Anderson and Dill was used in our study to measure the exposure level of violent video games. VVGE was a self-reporting questionnaire that contained five items. The participants were asked to list up to five of their favorite video games they usually play and then record their frequency of playing each game on a scale of 1–7 (with 1 denoting never play and 7 denoting almost addicted). The degree of violent content and violent graphics of each game was also rated with a seven-point Likert scale ranging from 1 to 7 (with 1 representing rarely, little or no violent content and little or no violent graphics, 7 denoting often, extremely violent content and extremely violent graphics). The VVGE score of each participant was computed by summing the rating of the violent content and violent graphics and multiplying it by the rating frequency of each game. Then, the VVGE score was divided by the listed numbers of games played by the participants to provide an overall score of VVGE (23). According to the overall score of VVGE, the exposure level of violent video games can be classified into four categories: no VVGE, low VVGE, medium VVGE, and high VVGE. Among them, no VVGE included participants who did not play video games and those who played video games but did not contain violence, low VVGE referred to participants whose exposure level to violent video games was below the 33rd percentile, medium VVGE referred to the participants whose exposure level to violent video games was between the 33rd to 66th percentile, and high VVGE referred to participants whose exposure level to violent video games was above the 66th percentile (24). The violent video game exposure questionnaire showed good reliability and validity in previous studies and was suitable for measuring the exposure level of violent video games among Chinese adolescents (25).

### Statistical analysis

Descriptive statistics were used to describe demographic characteristics of the participants and the prevalence of sub-types of school bullying victimization and perpetration [*n* (%)]. Then, the Spearman rank correlation was used to analyze the correlation of variables among the sub-types of school bullying, violent video game exposure, and Internet addiction.

For estimating the association of sub-types of school bullying with violent video game exposure, we performed the multiple logistic regression analysis with corresponding odds ratios (ORs) and corresponding 95% confidential intervals (CIs) in three different models. We considered the occurrence of different sub-types of school bullying as the dependent variable, and the degree of violent video game exposure of middle school students was taken as the independent variable. To reduce the potential confounders, the increasing degree of adjustment was established in the models. In model 1, no variables were adjusted; model 2 was adjusted for gender and grade. Based on the adjustment in model 2, model 3 was adjusted for smoking status, drinking status, number of friends, relationship between family members, relationship between themselves and their parents, frequency of communication with their parents, level of Internet addiction.

All data analyses were performed by using IBM SPSS 25.0, with the two-sided *P*-value < 0.05 as statistically significant.

## Results

### Demographic characteristics of the participants

Among the 1,992 participants, 1,096 (55.02%) were boys and 896 (44.98%) were girls with an average age of 15.84 ± 1.62 years. The percentage of senior high school students (53.72%) was slightly more than that for junior high school students (46.28%). Only 162 (8.13%) of the students were smokers, while alcohol users (2.06%) were fewer. A great number of students (84.04%) had more than three friends. Only 18.78% of the students were the only child in their families. In all, 1,398 (70.18%) were normal Internet users, 514 (25.80%) showed moderate Internet addictive behaviors, while only 31 (1.56%) of the students showed severe Internet addictive behaviors. The distribution of family income, family structure, family member's relationship, parent–child relationship, and frequency of communication with parents is shown in Table 1.

**Table 1:** Demographic characteristics and the prevalence of sub-types of school bullying and VVGE of the participants.

**Variables**	***N***	**%**
**Gender**		
Boy	1,096	55.02
Girl	896	44.98
**Grade**		
7^th^	320	16.06
8^th^	602	30.22
10^th^	486	24.40
11^th^	584	29.32
**Smoking status**		
Yes	162	8.13
No	1,830	91.87
**Drinking status**		
Yes	41	2.06
No	1,951	97.94
**Number of friends**		
1 or less	83	4.16
2–3	235	11.80
More than 3	1,674	84.04
**Only child**		
Yes	374	18.78
No	1,618	81.22
**Internet addiction** ^**a**^		
Normal Internet users	1,398	70.18
Moderate addictive behaviors	514	25.80
Severe addictive behaviors	31	1.56
**Family affluence**		
Poor	119	5.97
Average	1,467	73.65
Rich	406	20.38
**Family structure**		
Two-parent family	1,886	94.68
Single-parent family	73	3.66
Reconstituted family	33	1.66
**Family member's relationship**		
Harmony	1,484	74.50
General	442	22.19
Always conflict	66	3.31
**Parent-child relationship**		
Never fight	315	15.81
Occasional fight	1,588	79.72
Always fight	89	4.47
**Frequency of communication with parents**		
Seldom	132	6.63
Occasional	821	41.21
Regular	1,039	52.16
**Sub-types of school bullying victimization** ^b^		
Physical	361	18.12
Verbal	1,202	60.34
Relational	234	11.75
Cyber	240	12.05
**Sub-types of school bullying perpetration** ^b^		
Physical	331	16.62
Verbal	1,088	54.62
Relational	428	21.49
Cyber	164	8.23
**Violent video game exposure**		
No VVGE	552	27.70
Low-level VVGE	480	24.10
Medium-level VVGE	482	24.20
High-level VVGE	478	24.00
**Total**	1,992	100.00

a Forty-nine participants reported never used the Internet.

b The co-occurrence of different sub-types of school bullying.

### Prevalence of sub-types of school bullying

In this study, 361 (18.12%) of the 1,992 students were victimized by physical school bullying and 1,202 (60.34%) of them were victimized by verbal school bullying in the past year. Only 234 (11.75%) students were victimized by relational school bullying, and 240 (12.05%) students were victimized by cyber school bullying in the past year. In terms of the bullying perpetration behaviors, the reported prevalence of the four sub-types of school bullying during the last year was 16.62% (physical), 54.62% (verbal), 21.49% (relational), and 8.23% (cyber; Table 1).

### Prevalence of violent video game exposure

Of the students, 552 students had never played violent video games, and the prevalence of no violent video game exposure (no VVGE) was 27.70%; 480 (24.10%) of the students reported low-level violent video game exposure (low-level VVGE); 482 (24.20%) reported medium-level violent video game exposure (medium-level VVGE); and 478 (24.00%) reported high-level violent video game exposure (high-level VVGE; Table 1).

### Associations between sub-types of school bullying and violent video game exposure

The Spearman correlations between sub-types of school bullying and violent video game exposure are shown in Table 2. In this study, four types of school bullying victimization or perpetration were significantly positively associated, with the correlation coefficient ranging from 0.15–0.32 (victimization) to 0.24–0.37 (perpetration). There was also a significant positive correlation between victimization and perpetration among all the sub-types of school bullying in the students (*r*_s_ = 0.13–0.78, *P* < 0.01). The no VVGE and low-level VVGE students were negatively associated with the sub-types of school bullying victimization and perpetration (*r*_s_ = −0.07 to −0.18, *P* < 0.05), while the medium-level VVGE and high-level VVGE students were positively associated with the sub-types of school bullying victimization and perpetration (*r*_s_ = 0.05~0.18, *P* < 0.05). In addition, the students who were normal Internet users were statistically negatively correlated to all the sub-types of school bullying victimization and perpetration (*r*_s_= −0.08 to −0.14, *P* < 0.05). For the students showing moderate addiction behaviors or severe addictive behaviors, its correlation with the sub-types of school bullying victimization and perpetration was positive (*r*_s_ = 0.05–0.15, *P* < 0.05).

**Table 2:** Correlations among sub-types of school bullying and violent video game exposure.

**Variables**	**1**	**2**	**3**	**4**	**5**	**6**	**7**	**8**	**9**	**10**	**11**	**12**	**13**	**14**	**15**
1.Physical victimization	–														
2.Verbal victimization	0.29**	–													
3.Relational victimization	0.27**	0.23**	–												
4.Cyber victimization	0.15**	0.26**	0.32**	–											
5.Physical perpetration	0.53**	0.27**	0.19**	0.13**	–										
6.Verbal perpetration	0.27**	0.78**	0.17**	0.20**	0.29**	–									
7.Relational perpetration	0.27**	0.34**	0.23**	0.25**	0.28**	0.37**	–								
8.Cyber perpetration	0.20**	0.21**	0.15**	0.27**	0.25**	0.24**	0.30**	–							
9.No VVGE	−0.16**	−0.15**	−0.10**	−0.08**	−0.15**	−0.18**	−0.12**	−0.11**	–						
10.Low-level VVGE	−0.07**	−0.07**	0.02	−0.01	−0.08**	−0.07**	−0.02	−0.07**	−0.35**	–					
11.Medium-level VVGE	0.08**	0.13**	0.06**	0.06*	0.06**	0.14**	0.05*	0.06**	−0.35**	−0.32**	–				
12.High-level VVGE	0.15**	0.10**	0.03	0.03	0.18**	0.12**	0.10**	0.12**	−0.35**	−0.32**	−0.32**	–			
13.Normal Internet usage	−0.04	−0.11**	−0.13**	−0.12**	−0.08**	−0.12**	−0.12**	−0.14**	−0.01	0.06**	0.03	−0.09**	–		
14.Moderate signs of addiction	0.04	0.13**	0.12**	0.12**	0.08**	0.13**	0.13**	0.15**	−0.02	−0.05*	−0.03	0.10**	−0.91**	–	
15.Severe addictive behaviors	0.04	0.01	0.07**	0.04	0.05*	0.03	0.04	0.05*	0.01	−0.04	0.03	0.01	−0.20**	−0.07**	–

^*^P < 0.05. ^**^P < 0.01.

The results of multiple logistic regression analysis with the degree of violent video game exposure regarded as a continuous variable are displayed in Table 3. In model 1, the prevalence rates of physical victimization, physical perpetration, relational perpetration, and cyber perpetration significantly increased with the increasing degrees of violent video game exposure (*P* for trend < 0.001). The highest ORs of physical victimization, physical perpetration, relational perpetration, and cyber perpetration risk for high-level VVGE were 4.329 (95% CI 3.016–6.215), 4.779 (95% CI 3.293–6.935), 2.529 (95% CI 1.847–3.463), and 4.573 (95% CI 2.704–7.734), respectively. A slight decrease in high-level VVGE was observed, while the prevalence rate of verbal victimization, relational victimization, cyber victimization, and verbal perpetration significantly increased with the increasing degree of violent video game exposure from no VVGE to medium-level VVGE.

**Table 3:** Multiple logistic regression analysis of sub-types of school bullying and violent video game exposure [OR (95% CI)].

**Variables**	**The degree of violent video game exposure**				***P* for trend**
	**No VVGE**	**Low-level VVGE**	**Medium-level VVGE**	**High-level VVGE**	
**Model 1** ^**a**^					
Physical victimization (ref. = no)	1.00	1.723 (1.156, 2.569)	3.447 (2.387, 4.977)	4.329 (3.016, 6.215)	< 0.001
Verbal victimization (ref. = no)	1.00	1.281 (1.003, 1.637)	2.708 (2.090, 3.508)	2.397 (1.856, 3.096)	< 0.001
Relational victimization (ref. = no)	1.00	2.150 (1.392, 3.323)	2.679 (1.756, 4.088)	2.284 (1.483, 3.517)	< 0.001
Cyber victimization (ref. = no)	1.00	1.636 (1.076, 2.488)	2.202 (1.476, 3.287)	1.979 (1.318, 2.974)	0.001
Physical perpetration (ref. = no)	1.00	1.571 (1.031, 2.396)	3.139 (2.137, 4.610)	4.779 (3.293, 6.935)	< 0.001
Verbal perpetration (ref. = no)	1.00	1.381 (1.079, 1.768)	2.919 (2.264, 3.765)	2.702 (2.098, 3.481)	< 0.001
Relational perpetration (ref. = no)	1.00	1.569 (1.127, 2.186)	2.155 (1.567, 2.964)	2.529 (1.847, 3.463)	< 0.001
Cyber perpetration (ref. = no)	1.00	1.476 (0.798, 2.730)	3.539 (2.067, 6.062)	4.573 (2.704, 7.734)	< 0.001
**Model 2** ^**b**^					
Physical victimization (ref. = no)	1.00	1.430 (0.949, 2.155)	2.288 (1.558, 3.360)	2.524 (1.718, 3.707)	< 0.001
Verbal victimization (ref. = no)	1.00	1.230 (0.960, 1.577)	2.491 (1.905, 3.257)	2.137 (1.625, 2.810)	< 0.001
Relational victimization (ref. = no)	1.00	2.177 (1.405, 3.373)	2.784 (1.800, 4.306)	2.451 (1.551, 3.875)	< 0.001
Cyber victimization (ref. = no)	1.00	1.655 (1.085, 2.524)	2.343 (1.548, 3.546)	2.226 (1.442, 3.438)	< 0.001
Physical perpetration (ref. = no)	1.00	1.331 (0.866, 2.046)	2.209 (1.482, 3.292)	3.016 (2.037, 4.466)	< 0.001
Verbal perpetration (ref. = no)	1.00	1.313 (1.023, 1.685)	2.619 (2.014, 3.406)	2.315 (1.768, 3.032)	< 0.001
Relational perpetration (ref. = no)	1.00	1.508 (1.079, 2.106)	1.979 (1.423, 2.753)	2.285 (1.634, 3.195)	< 0.001
Cyber perpetration (ref. = no)	1.00	1.364 (0.734, 2.533)	2.932 (1.687, 5.097)	3.478 (2.007, 6.027)	< 0.001
**Model 3** ^**c**^					
Physical victimization (ref. = no)	1.00	1.552 (1.015, 2.374)	2.209 (1.482, 3.294)	2.251 (1.501, 3.375)	< 0.001
Verbal victimization (ref. = no)	1.00	1.225 (0.944, 1.590)	2.326 (1.756, 3.080)	1.770 (1.326, 2.363)	< 0.001
Relational victimization (ref. = no)	1.00	2.238 (1.414, 3.540)	2.566 (1.622, 4.060)	1.986 (1.222, 3.227)	0.001
Cyber victimization (ref. = no)	1.00	1.768 (1.140, 2.740)	2.169 (1.406, 3.348)	1.786 (1.130, 2.823)	0.006
Physical perpetration (ref. = no)	1.00	1.476 (0.942, 2.311)	2.092 (1.379, 3.175)	2.554 (1.685, 3.870)	< 0.001
Verbal perpetration (ref. = no)	1.00	1.372 (1.057, 1.782)	2.413 (1.834, 3.175)	1.903 (1.432, 2.529)	< 0.001
Relational perpetration (ref. = no)	1.00	1.515 (1.071, 2.142)	1.729 (1.228, 2.435)	1.693 (1.191, 2.409)	0.009
Cyber perpetration (ref. = no)	1.00	1.520 (0.802, 2.880)	2.597 (1.464, 4.609)	2.520 (1.421, 4.468)	0.003

a Model 1 was not adjusted.

b Model 2 was adjusted for gender and grade.

c Model 3 was further adjusted for smoking status, drinking status, numbers of friends, family member's relationship, relationship with parents, frequency of communication with parents, and Internet addiction level.

In model 2, after controlling for confounding variables of gender and grade, the results were similar to those of model 1. The highest adjusted ORs of physical victimization, physical perpetration, relational perpetration, and cyber perpetration risk were at high-level VVGE compared with no VVGE were 2.524 (95% CI 1.718–3.707), 3.016 (95% CI 2.037–4.466), 2.285 (95% CI 1.634–3.195), and 3.478 (95% CI 2.007–6.027), respectively. After further adjusting potential confounders in model 3, the risk of physical victimization and physical perpetration significantly increased with the increasing degree of violent video game exposure (*P* for trend < 0.001), with the highest adjusted ORs being 2.251 (95% CI 1.501–3.375) and 2.554 (95% CI 1.685–3.870), when comparing high-level VVGE with no VVGE. While the risk of verbal victimization, relational victimization, cyber victimization, verbal perpetration, relational perpetration, and cyber perpetration was significantly increased with the increasing degree of violent video game exposure from no VVGE to medium-level VVGE, students who reported high-level VVGE had a lower risk of types of school bullying compared with those who reported medium-level VVGE (Table 3).

## Discussion

The present observational study investigates the relationship between violent video game exposure and school bullying behaviors in Chinese adolescents. Our results showed that both school bullying behaviors and violent video game exposure among Chinese adolescents were major problems. Violent video game exposure was associated with school bullying behaviors in Chinese adolescents.

The main finding of our study was that adolescents in no VVGE and low-level VVGE categories were negatively associated with different sub-types (physical, verbal, relational, and cyber) of school bullying victimization and perpetration, while the adolescents in medium-level VVGE and high-level VVGE categories were positively associated with the sub-types of school bullying victimization and perpetration. These results suggested that the gaming behavior of adolescents who were exposed to a medium or high level of VVGs could not be changed unless enforcing gaming frequency restrictions in the anti-school bullying program, which was also consistent with the previous study findings (26). However, a balanced perspective should be adopted along with the program since normal Internet adolescent's users or adolescents under no VVGE or low-level VVGE categories were negatively associated with school bullying behaviors, which indicated the possible benefits of game playing (27). Notably, our results among these weak correlations between different sub-types of school bullying and VVGE were also in line with previous studies (26, 28, 29).

With the increased degree of violent video game exposure from no VVGE to medium-level VVGE, the risk of different sub-types of school bullying showed a significant increase. More importantly, the current study found that the risk of physical victimization and physical perpetration was significantly increased with the increasing degree of violent video game exposure from no VVGE to high-level VVGE. Our finding is consistent with previous studies that adolescents who played more VVGs became more physically aggressive (30), and we also extended the existing studies by reporting that those who played more VVGs were at a higher risk of being physical bullying victims in school. The dose–response association between the degree of VVGE and physical school bullying behaviors including physical victimization and perpetration was also identified in our study. The reason why adolescents who were exposed to more VVGs were more likely to be physical victims of school bullying could be explained as follows: on the one hand, playing VVGs could be used as a way of emotional regulation to escape from the pain caused by physical school bullying in adolescents (10). On the other hand, adolescents might also identify themselves as physical bully victims, and the co-occurrence of physical bullying perpetration and victimization might be found among the students who played a lot of VVGs. Unlikely, the same dose–response relationship was not found in other sub-groups since the risk of school bullying only continuously increased from no VVGE to medium-level VVGE as VVGE is more associated with physical aggression behaviors or physical school bullying behaviors were more directly to recollect than other sub-types in the survey (26).

The general aggression model (GAM) was the most used method in the explanation of the association between adolescents' greater exposure to VVGs and physical school bullying perpetrators (11). In GAM, Anderson and Bushman believed that the youth repeatedly exposed to VVGs developed aggressive beliefs, perceptual schemata, and aggressive behaviors automatically, which cultivates an aggressive personality in the long term. This, in turn, after the environmental stimulation available in school, adolescents who were exposed to more VVGs would become more aggressive and thus perpetrate physical school bullying behaviors toward their peers (11, 31). Furthermore, some other theories such as the information processing model presented by Huesmann explained that the youth could become aggressive when acquiring aggressive scripts, which were stable and resistant to change once established (32). VVGE was one of the ways that adolescents acquire aggressive scripts, which, once acquired and maintained, could lead to physical bullying perpetration behavior in them.

Over the different sub-groups of school bullying, traditional school bullying behaviors, such as physical, verbal, and relational school bullying, were positively associated with cyberbullying, respectively. These results that suggest that traditional bullying overlapped with cyberbullying was also confirmed in previous studies (7, 33, 34), and since the distinguishing feature of cyberbullying was anonymous and feasible, cyberbullying could be an extended form of traditional school bullying (35). School bullying intervention programs should address traditional bullying and cyberbullying altogether. Most importantly, all sub-types of school bullying victimization and perpetration behaviors showed a mutually positive association. This contributed to the reason why the association between the degree of VVGE and any type of school bullying victimization was similar to that with any type of school bullying perpetration, suggesting that adolescents who were at risk of being exposed to VVGs are more vulnerable to become a school bullying perpetrator, victim, or both.

The prevalence of sub-types of school bullying among Chinese adolescents in the study was at a high level and between the rates in previous studies (1, 5, 36), which would account for the disunified school bullying measurements in different studies (22, 36). Cultural, regional, and economic variation could also be the factors influencing the prevalence observed in different studies on school bullying among Chinese adolescents (36, 37). School bullying, as one of the serious public health problems in adolescents, effective anti-school bullying interventions are needed in this population through school and family. When we examined the relationship between school bullying and VVGE in Chinese adolescents, we also found that moderate Internet addiction was correlated with both school bullying and VVGE, which indicated that Internet addiction might be a potential mediator in the association between school bullying and VVGE among Chinese adolescents and that VVGE might lead to Internet addiction among the youth, while adolescents addicted to the Internet were most likely to experience school bullying behaviors (38). Therefore, adolescents with Internet addiction signs should not be overlooked in the early stage of school bullying prevention programs or in preventing excessive video gaming of adolescents.

There are several limitations that need to be addressed in this study. First, through the current observational study, it was difficult to draw causal associations between different sub-types of school bullying and VVGE among middle school students. Strong evidence from further studies with a longitudinal design exploring these relationships could be of benefit. Second, we could not avoid the self-reporting bias and recall bias since some information (e.g., family affluence, family member's relationship, experiences of school bullying in the past year, and students' favorite video games) was collected by questionnaires from the participants. Moreover, we did not consider other potential confounding variables, such as overweight, obesity, sexual orientation, physical disability, and school environment factors, which may moderate the association between school bullying and VVGE in adolescents (39–42). Finally, our study identifying the association between school bullying and VVGE might be constrained by the sample size collected from only one province in China; therefore, future studies with more participants in different regions of China through a multi-center design could be more effective.

Despite the limitations of the study, our findings provide a number of practical implications for school bullying prevention in the youth. First, our results indicated that school bullying was common among Chinese adolescents, and anti-school bullying prevention programs to reduce the prevalence of school bullying conducted both in the school and in family are essential for school bullying perpetrators and victims (43). At the same time, health education and promotion for adolescents who were involved in any type of school bullying are also needed (44). Second, our findings highlight the need for network regulations for teenagers as the associations between VVGE and school bullying behaviors, especially physical school bullying, were positive among Chinese adolescents. Furthermore, puzzle games and pro-social video games, instead of VVGs, are advocated for the gaming merchants in video game development (14). In addition, a longitudinal multi-center study with a larger population including more Chinese provinces and considering more potential confounders need to be carried out to examine how and why physical school bullying perpetration and victimization have distinct associations with VVGE among Chinese adolescents in future research.

## Conclusion

These findings highlight the specific association between different sub-types of school bullying involvement and violent video game exposure. This might be valuable information for school policymakers and researchers in developing new physical school bullying prevention and intervention programs based on network, especially VVG regulations among middle school students in China, as with the increased degree of violent video game exposure, the incidence of physical school bullying also increased. Multi-center longitudinal studies are also needed to identify the causal relationship between violent video game exposure and school bullying in Chinese adolescents.

## Data availability statement

The raw data supporting the conclusions of this article will be made available by the authors, without undue reservation.

## Ethics statement

The studies involving human participants were reviewed and approved by Shantou University Medical College. Written informed consent to participate in this study was provided by the participants' legal guardian/next of kin.

## Author contributions

YS designed the study, verified, analyzed data, and drafted the manuscript. ZY involved in data collection and data cleaning. LX contributed to data collection. LL supervised the study, accessed and verified data, revised the manuscript, and approved the final version. All authors have read and approved the final manuscript.

## Conflict of interest

The authors declare that the research was conducted in the absence of any commercial or financial relationships that could be construed as a potential conflict of interest.

## Publisher's note

All claims expressed in this article are solely those of the authors and do not necessarily represent those of their affiliated organizations, or those of the publisher, the editors and the reviewers. Any product that may be evaluated in this article, or claim that may be made by its manufacturer, is not guaranteed or endorsed by the publisher.
